# Clinical Evaluation of Oxidative Stress Markers in Patients with Long COVID During the Omicron Phase in Japan

**DOI:** 10.3390/antiox14091068

**Published:** 2025-08-30

**Authors:** Osamu Mese, Yuki Otsuka, Yasue Sakurada, Kazuki Tokumasu, Yoshiaki Soejima, Satoru Morita, Yasuhiro Nakano, Hiroyuki Honda, Akiko Eguchi, Sanae Fukuda, Junzo Nojima, Fumio Otsuka

**Affiliations:** 1 Department of General Medicine, Okayama University Graduate School of Medicine, Dentistry and Pharmaceutical Sciences, Okayama 700-8558, Japan; pded9h77@okayama-u.ac.jp (O.M.); otsuka@s.okayama-u.ac.jp (Y.O.); pzaf6h9w@s.okayama-u.ac.jp (Y.S.); tokumasu@okayama-u.ac.jp (K.T.); p32v0ja8@s.okayama-u.ac.jp (Y.S.); p2fb2mqg@s.okayama-u.ac.jp (S.M.); y-nakano@okayama-u.ac.jp (Y.N.); ppgf1hrd@s.okayama-u.ac.jp (H.H.); 2 Biobank Center, Mie University Hospital, Tsu 514-8507, Japan; akieguchi@med.mie-u.ac.jp; 3 Department of Gastroenterology and Hepatology, School of Medicine, Mie University, Tsu 514-8507, Japan; 4 Department of Health Welfare Sciences, Kansai University of Welfare Sciences, Kashiwara 582-0026, Japan; sfukuda@tamateyama.ac.jp; 5 Department of Laboratory Medicine, Yamaguchi University, Ube 755-8505, Japan; nojima-j@yamaguchi-u.ac.jp

**Keywords:** biological antioxidant potential (BAP), Coronavirus disease 2019 (COVID-19), diacron-reactive oxygen metabolites (d-ROM), Long COVID, oxidative stress index (OSI)

## Abstract

To characterize changes in markers of oxidative stress for the clinical evaluation of patients with long COVID, we assessed oxidative stress and antioxidant activity based on serum samples from patients who visited our clinic between May and November 2024. Seventy-seven patients with long COVID (41 [53%] females and 36 [47%] males; median age, 44 years) were included. Median [interquartile range] serum levels of diacron-reactive oxygen metabolites (d-ROM; CARR Unit), biological antioxidant potential (BAP; μmol/L), and oxidative stress index (OSI) were 533.8 [454.9–627.6], 2385.8 [2169.2–2558.1] and 2.0 [1.7–2.5], respectively. Levels of d-ROMs (579.8 vs. 462.2) and OSI (2.3 vs. 1.8), but not BAP (2403.4 vs. 2352.6), were significantly higher in females than in males. OSI levels positively correlated with age and body mass index, whereas BAP levels negatively correlated with these parameters. d-ROM and OSI levels were significantly associated with inflammatory markers, including C-reactive protein (CRP) and fibrinogen, whereas BAP levels were inversely correlated with CRP and ferritin levels. Notably, serum free thyroxine levels were negatively correlated with d-ROMs and OSI, whereas cortisol levels were positively correlated with d-ROMs. Among long COVID symptoms, patients reporting brain fog exhibited significantly higher OSI levels (2.2 vs. 1.8), particularly among females (d-ROMs: 625.6 vs. 513.0; OSI: 2.4 vs. 2.0). The optimal OSI cut-off values were determined to be 1.32 for distinguishing long COVID from healthy controls and 1.92 for identifying brain fog among patients with long COVID. These findings suggest that oxidative stress markers may serve as indicators for the presence or prediction of psycho-neurological symptoms associated with long COVID in a gender-dependent manner.

## 1. Introduction

Coronavirus disease 2019 (COVID-19) causes a wide variety of symptoms, not only during the acute phase following severe acute respiratory syndrome coronavirus 2 (SARS-CoV-2) infection, but also during the chronic phase, affecting up to 20% to 40% of patients [1,2,3,4]. This persistent illness, referred to as post-COVID-19 condition (PCC), post-acute sequelae of COVID-19 (PASC), or long COVID [5,6], manifests as a broad spectrum of symptoms, including fatigue, dyspnea, myalgia, arthralgia, and cognitive dysfunction, including concentration deficit. Long COVID typically begins within two to three months of initial infection and cannot be explained by an alternative diagnosis [7]. The pathophysiology of long COVID remains unclear, although various mechanisms, including microthrombus, persistent inflammation, autoimmunity, viral persistence, neurological damage, vascular dysfunction, and hypochondria, have been proposed [8,9,10].

Epidemiological data suggest that, partly due to the impact of vaccination, the overall risk of developing long COVID has decreased from early pandemic strains to the Omicron phase, both in Europe and in Japan [11,12,13,14]. Nevertheless, long COVID still occurs in approximately 10% of individuals infected with COVID-19 [11,13], indicating that it remains a significant public health concern. Furthermore, in Japan, the risk of cognitive symptoms, such as brain fog, appears to be relatively higher during the Omicron period compared to previous variants [15].

This complex mechanism suggests the need for multidisciplinary rather than single-modality therapeutic approaches. Patients with long COVID frequently report symptoms that resemble those of myalgic encephalomyelitis/chronic fatigue syndrome (ME/CFS) [16,17]. Degenerative conditions characterized by persistent multisystemic symptoms lasting longer than six months include pathological fatigue, mental and physical exhaustion, sleep disturbances, pain, autonomic dysfunction, and cognitive impairment [18,19]. Although some screening tools for symptom differentiation have been established, and elevated serum ferritin has been proposed as a possible biomarker for ME/CFS in patients with long COVID, no definitive biomarkers have been identified to date [11,20,21,22]. Various endocrine factors, including those related to the hypothalamo–pituitary–adrenal (HPA) axis, sex steroids, and thyroid hormones, may also be involved in the development and persistence of some of the long COVID symptoms [23,24,25,26,27].

In the present study, we aimed to investigate whether markers of oxidative stress and/or antioxidant activity, which are frequently altered in infectious diseases, could serve as potential monitoring biomarkers for distinguishing subgroups of patients with long COVID. Furthermore, we assessed whether these markers could help exclude cases that mimic long COVID but are in fact false positives. There have been only a few reports evaluating oxidative stress markers in patients with COVID-19, primarily during the acute phase. These studies aimed to assess clinical management strategies of inflammatory conditions and disease severity, facilitate the stratification of at-risk individuals, and optimize the allocation of public health resources [28,29]. However, fundamental data on patients with long COVID remain lacking. We therefore assessed oxidative stress markers, including serum levels of diacron-reactive oxygen metabolites (d-ROMs) and biological antioxidant potential (BAP), and calculated oxidative stress index (OSI) based on these values [30,31,32]. We then examined how these markers were associated with clinical background and individual patient characteristics.

## 2. Patients and Methods

### 2.1. Study Design and Participants

This retrospective observational study was conducted at Okayama University Hospital, a tertiary care center, between 22 May and 28 November 2024. We included patients with long COVID who were referred to the COVID-19 aftercare outpatient clinic (CAC) by local doctors who provided written informed consent. The CAC, located within the Department of General Medicine at the Okayama University Hospital, serves as a regional hub for long COVID care in Western Japan [33].

In this study, a healthy control was compared to patients with long COVID. The control group consisted of 312 healthy individuals recruited under strict exclusion criteria based on lifestyle surveys, including no smoking, no heavy drinking, absence of metabolic syndrome, no pregnancy, and no regular medication use [31,32]. None of these individuals had a history of thrombosis, obstetric complications, or abnormal findings on physical or laboratory examinations [31,32].

### 2.2. Inclusion and Exclusion Criteria

All adult patients (≥18 years old) with long COVID who visited the CAC were eligible. Patients were included if they were invited by their attending physician and if they consented voluntarily. Patients who did not undergo blood tests were excluded. Acute diagnosis of COVID-19 was performed by their own local doctors who had referred to our hospital based on antigen examination or PCR testing for COVID-19. Long COVID was defined as the presence of symptoms that persisted for >4 weeks after the onset of COVID-19.

### 2.3. Variables and Data Collection

Data were extracted from electronic medical records and included demographics (age, sex, and body mass index [BMI]), smoking status, duration from COVID-19 onset to first CAC visit, history of COVID-19 vaccination, clinical symptoms and courses, laboratory results, and treatment details. Acute COVID-19 severity was defined based on the Japanese classification criteria, which consider oxygen saturation, respiratory symptoms, and pulmonary involvement [34]. Individual interviews were carefully conducted to identify long COVID–related symptoms. Chief complaints were compiled and classified for study purposes by consensus between three researchers (MO, YO, and YS). Brain fog was defined as subjective difficulty concentrating, mental slowness, or a feeling of being “spaced out”, particularly when such symptoms impaired cognitive function or were accompanied by headache [35].

### 2.4. Measurements of Laboratory Markers

Blood samples were basically collected with patients in the seated position during late morning time at their initial visit to the CAC outpatient clinic. Routine laboratory tests were performed using automated analyzers at the central laboratory of Okayama University Hospital. We analyzed inflammatory (C-reactive protein [CRP], fibrinogen, and ferritin) and endocrine (free thyroxine and cortisol) markers in the patients’ sera. Hormone levels were measured using the Cobas 8000 system (F. Hoffmann-La Roche AG, Basel, Switzerland) with electrochemiluminescence immunoassay kits: Elecsys FT4 III and Elecsys Cortisol II (F. Hoffmann-La Roche AG) [27].

### 2.5. Measurement of d-ROMs, BAP, and OSI

To assess oxidative and antioxidative statuses, a maximum of 5 milliliters of additional blood were collected from each patient simultaneously with other laboratory tests. Levels of d-ROM and BAP were measured simultaneously in serum, as described previously [30,31,32]. Blood samples from both control groups were collected at rest under comparable conditions. Oxidative stress was measured using d-ROMs (Diacron International, Grosseto, Italy), and antioxidant capacity was assessed using BAP (Diacron International) on an AU480 automated analyzer (Beckman Coulter, Tokyo, Japan) [30]. In the d-ROM test [36,37], hydroperoxides in serum react via the Fenton reaction to produce alkoxyl and peroxyl radicals, which oxidize N,N-diethyl-p-phenylenediamine to generate a pink chromogen measured photometrically at 505 nm. d-ROMs are expressed in Carratelli Units (1 CARR U = 0.08 mg H_2_O_2_/dL) [37]. In the BAP test [38], serum decolorizes a solution of ferric chloride and thiocyanate derivative, with absorbance measured at 505 nm. The oxidative stress index (OSI) was calculated as follows: OSI = C × (d-ROMs/BAP), where C is a standardization coefficient set to make the mean OSI of healthy controls equal to 1.0 [30,31,32].

### 2.6. Statistical Analysis

All statistical analyses were conducted using Stata/SE19 (StataCorp LLC, College Station, TX, USA). Pearson’s χ2 test was used for categorical variables. The Mann–Whitney U test was used for continuous variables with non-normal distributions, as confirmed by the skewness–kurtosis test. Linear regression analysis and Spearman’s rank correlation were used to analyze associations between variables. Receiver operating characteristic (ROC) curve analysis was performed to evaluate the diagnostic performance of continuous variables, and optimal cut-off values were determined using Youden’s J statistic. In addition, multivariable linear regression analyses were conducted within the patient group to account for patient background factors, with robust standard errors applied. A *p*-value < 0.05 was considered statistically significant.

### 2.7. Ethical Approval

This study was approved by the Ethical Committee of Mie University (No. H2023-171) and Okayama University Hospital (No. 2311-020) and was conducted in accordance with the Declaration of Helsinki. Written informed consent was obtained from all the participants.

## 3. Results

As shown in Table 1, 77 patients with long COVID (41 [53%] females and 36 [47%] males; median age: 44 years) who visited our outpatient clinic were included in the present study. Smoking habits were reported by 30% (23 cases) of the patients, and 94% (72 cases) of them experienced only mild severity during the acute phase of COVID-19 (Table 1). Among the participants, 64% (49 cases) visited the hospital >90 days after the onset of infection of SARS-CoV-2, 16% (12 cases) had received no COVID-19 vaccination, none had received only one dose, and 84% (65 cases) had received at least two doses of COVID-19 vaccinations (Table 1).

As shown in Table 2, in 77 patients with long COVID, the median [interquartile range (IQR)] serum levels of d-ROMs (CARR Unit), BAP (μmol/L), and OSI were 533.8 [454.9–627.6], 2385.8 [2169.2–2558.1], and 2.0 [1.7–2.5], respectively (Table 2). Reference data from a healthy population (control group), obtained using the same methods, have been reported in previous studies, as shown in Table 2 [31,32]. The control group had a mean age of 36.7 years (standard deviation 8.8), consisting of 164 women and 148 men. Referential data are shown as median [IQR] values from healthy controls. Median [IQR] d-ROM, BAP, and OSI values in the 312 healthy cases were 287.4 [252.8–314.5] CARR Units, 2545.7 [2503.1–2583.5] mol/L, 1.0 [0.9–1.1], respectively [31,32] (Table 2). On the basis of the referential data, d-ROM and OSI values in the current 77 patients with long COVID, including 53% female cases, were significantly higher (*p* < 0.01) than those previously reported in the healthy population, while the levels of BAP in the patients were also significantly lower (*p* < 0.01) than the healthy controls (Table 2). Using the same measurement methods in a separate cohort of 12 healthy female participants (mean age 20.4 ± 0.5 years), pre-experimental acute fatigue measurements were as follows (mean ± standard deviation): d-ROMs, 303.4 ± 24.4 CARR Units; BAP, 2371.8 ± 77.6 μmol/L; OSI, 1.13 ± 0.07 [30]. Thus, the present data on the oxidative stress markers differed apparently compared to the healthy controls.

In addition, there were no significant differences in oxidative stress markers between individuals with (65 cases) and without (12 cases) a history of two and more vaccinations (Table 3).

**Figure 1:**
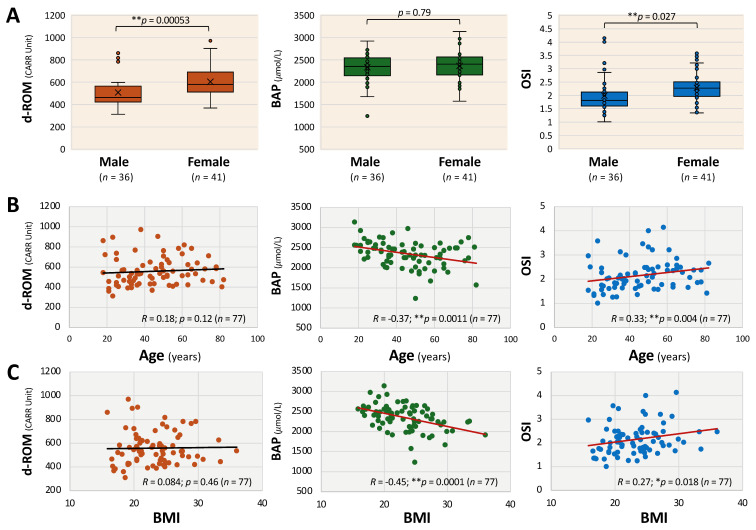
Characteristics of oxidative stress markers by sex, age, and BMI in patients with long COVID. (**A**) Box-and-whisker plots showing sex-dependent differences in diacron-reactive oxygen metabolites (d-ROMs), biological antioxidant potential (BAP), and oxidative stress index (OSI). Boxes represent the interquartile range, horizontal bars indicate the median, and “×” denotes the mean. (**B**) Scatter plots showing correlations between oxidative stress markers and patient age. (**C**) Scatter plots showing correlations between oxidative stress markers and body mass index (BMI). The Mann–Whitney U test was used for statistical comparisons. Linear regression analysis and Spearman’s rank correlation coefficients were used for statistical analysis. ** p < 0.01 and * p < 0.05 indicate statistical significance. Statistically significant regression lines are shown in red.

As shown in Figure 1, d-ROM (median: 579.8 vs. 462.2, ** *p* < 0.01) and OSI (median: 2.3 vs. 1.8, * *p* < 0.05) levels were significantly higher in female than in male patients with long COVID, while BAP levels (median: 2403.4 vs. 2352.6) did not significantly differ by sex (Figure 1A). OSI levels positively correlated with patient age (*R* = 0.33; ** *p* < 0.01) and BMI (*R* = 0.27; * *p* < 0.05), whereas BAP levels negatively correlated with age (*R*= −0.37; ** *p* < 0.01) and BMI (*R* = −0.45; ** *p* < 0.01) (Figure 1B,C). The levels of d-ROM were not significantly correlated with the patients’ age or BMI. In multivariable linear regression models restricted to patients with long COVID (Table 4), OSI was not significantly associated with age (*β* = 0.007; *p* = 0.063). Male sex was associated with lower OSI values compared to female sex (*β* = −0.322; *p* < 0.05), and BMI was positively associated with OSI (*β* = 0.032; *p* < 0.05). These findings indicate that sex and BMI may modestly influence OSI, whereas age is not a significant predictor.

**Figure 2:**
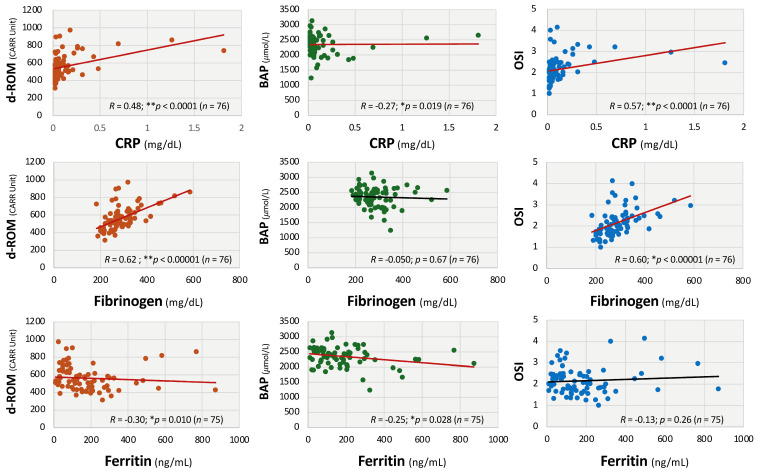
Correlations between oxidative stress markers and inflammatory markers in patients with long COVID. Linear regression analysis and the Spearman’s rank correlation were used to assess relationships between diacron-reactive oxygen (d-ROMs), oxidative stress index (OSI), and inflammatory markers, including C-reactive protein (CRP), fibrinogen, and ferritin. Linear regression analysis and Spearman’s rank correlation coefficients were used for statistical analysis. ** p < 0.01 and * p < 0.05 indicate statistical significance. Statistically significant regression lines are shown in red.

**Figure 3:**
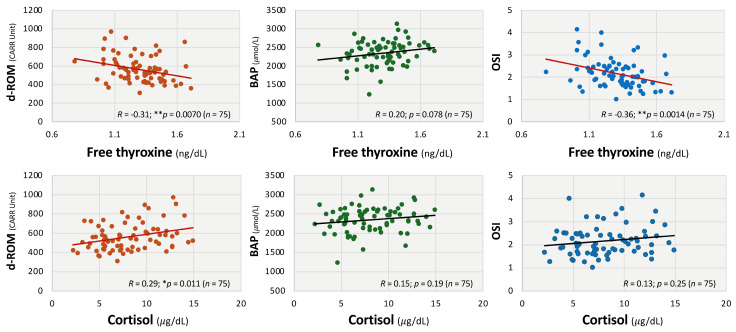
Correlations between oxidative stress markers and endocrine parameters in patients with long COVID. Correlations were evaluated between diacron-reactive oxygen metabolites (d-ROMs) and oxidative stress index (OSI) and endocrine factors, including serum free thyroxine and cortisol levels. Linear regression analysis and Spearman’s rank correlation coefficients were used for statistical analysis. ** p < 0.01 and * p < 0.05 indicates statistical significance. Statistically significant regression lines are shown in red.

As shown in Figure 2, d-ROM and OSI were significantly associated with inflammatory markers: CRP (d-ROM: *R* = 0.48; ** *p* < 0.01; OSI: *R* = 0.57; ** *p* < 0.01) and fibrinogen (d-ROM: *R* = 0.62; ** *p* < 0.01; OSI: *R* = 0.60; ** *p* < 0.01), while BAP showed negative correlations with CRP (*R* = −0.27; * *p* < 0.05) and ferritin (*R* = −0.25; * *p* < 0.05). In addition, a negative correlation between d-ROM and ferritin (*R* = −0.30; * *p* < 0.05) was observed (Figure 2). There were no significant correlations between the levels of BAP and fibrinogen and those between OSI and ferritin. From the endocrinological aspect, serum free thyroxine levels were negatively correlated with d-ROMs (*R* = −0.31; ** *p* < 0.01) and OSI (*R* = −0.36; ** *p* < 0.01), as shown in Figure 3, whereas serum cortisol levels were positively correlated with d-ROMs (*R* = 0.29; * *p* < 0.05). On the other hand, BAP levels were not significantly correlated with serum levels of free thyroxine and cortisol (Figure 3).

**Figure 4:**
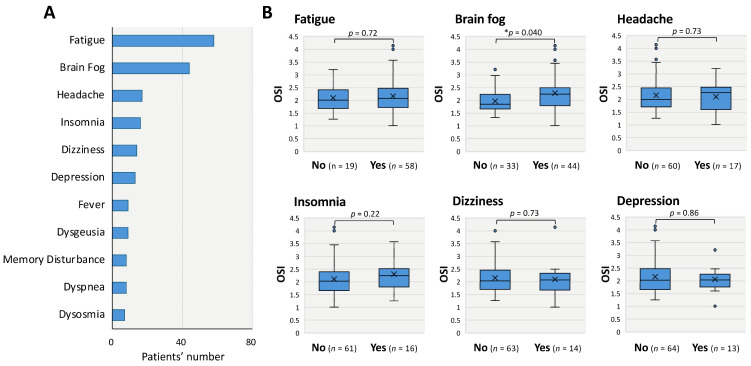
Associations between long COVID symptoms and oxidative stress index (OSI) in patients with long COVID. Number of patients who experienced each long COVID symptom (**A**) and the levels of oxidative stress index (OSI) by the presence of each symptom (**B**) are shown. Boxes represent the interquartile range, horizontal bars indicate the median, and “×” denotes the mean. The Mann–Whitney U test was used for statistical analysis. * p < 0.05 indicates statistical significance.

As shown in Figure 4, the clinical associations between the levels of oxidation markers and clinical characteristics were examined. Among the symptoms related to long COVID, the six most frequently reported were fatigue (75%), brain fog (57%), headache (22%), insomnia (21%), dizziness (18%), and depression (17%) (Figure 4A). As other minor symptoms, fever, dysgeusia, memory disturbance, dyspnea, and dysosmia were also included. To assess the utility of oxidative stress markers in evaluating these symptoms, OSI levels were compared between patients with and without each symptom listed in Figure 4A. As a result, only the patients’ group with brain fog exhibited significantly higher OSI levels than those without brain fog (median: 2.2 vs. 1.8, * *p* < 0.05) (Figure 4B). On the contrary, there were no significant differences in OSI data between patients with and without each representative symptoms: fatigue (median: 2.1 vs. 2.0), headache (2.3 vs. 2.0), insomnia (2.3 vs. 2.0), dizziness (2.1 vs. 2.0), and depression (2.0 vs. 2.0) (Figure 4B).

**Figure 5:**
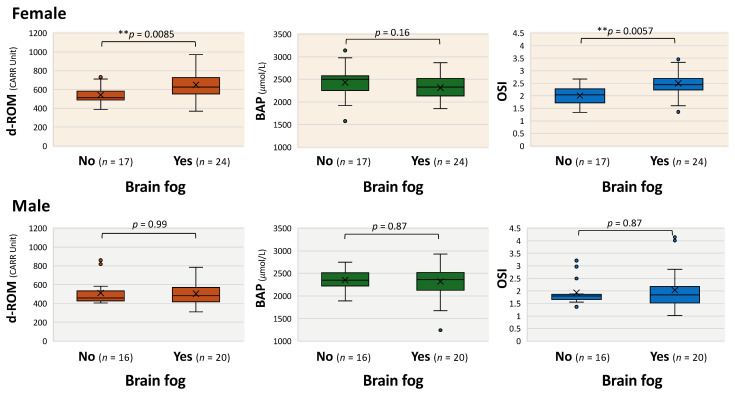
Characteristics of oxidative stress markers in patients with long COVID complaining of brain fog. Box-and-whisker plots illustrating sex-dependent differences in diacron-reactive oxygen metabolites (d-ROMs), biological antioxidant potential (BAP), and oxidative stress index (OSI) in patients with and/or without brain fog. Boxes represent the interquartile range, horizontal bars indicate the median, and “×” denotes the mean. The Mann–Whitney U test was used for statistical analysis; ** p < 0.01 indicates statistical significance.

Finally, to examine sex-specific associations, we compared oxidative markers with or without brain fog in female and male patients. Of note, in female patients, d-ROM and OSI levels were significantly higher in those with brain fog than in those without brain fog (median d-ROM: 625.6 vs. 513.0, ** *p* < 0.01; BAP: 2329.6 vs. 2498.8; OSI: 2.4 vs. 2.0, ** *p* < 0.01), whereas no significant difference was observed between the groups with and without brain fog in male patients (median: d-ROM: 480.9 vs. 456.6; BAP: 2363.6 vs. 2350.7; OSI: 1.8 vs. 1.8) (Figure 5). These findings suggest that oxidative stress markers may be useful for identifying or predicting brain fog in long COVID, particularly in a sex-dependent manner.

**Figure 6:**
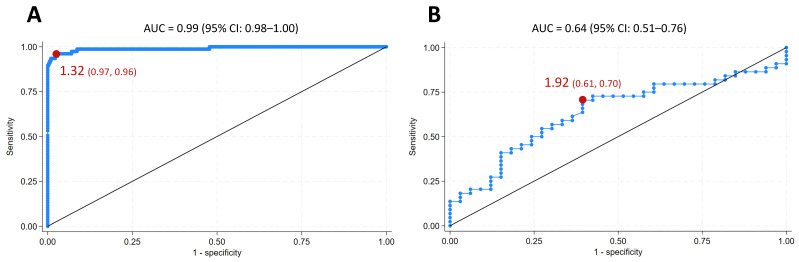
Receiver operating characteristic (ROC) curve analysis comparing the oxidative stress index (OSI) of healthy controls and the present patients with long COVID. ROC curve analysis was performed for (**A**) comparing the present patients with long COVID (77 cases) and healthy controls (312 cases) and (**B**) discriminating patients with brain fog (44 cases) among patients with long COVID, to evaluate the diagnostic performance of continuous variables of OSI, and the optimal cut-off values were determined using Youden’s J statistic.

As shown in Figure 6, ROC curve analysis of OSI levels was performed to compare our patients with long COVID to healthy controls. Figure 6A demonstrates an area under the curve (AUC) of 0.99 (95% Confidence Interval, 0.98–1.00), indicating a high discriminative ability for the diagnosis of long COVID. Using Youden’s J statistic, the optimal cut-off value for OSI was 1.32, which yielded a sensitivity of 0.96 and a specificity of 0.97. Moreover, as shown in Figure 6B, ROC analysis was performed to discriminate patients with brain fog among those with long COVID, in which the AUC was 0.64 (95% CI, 0.51–0.76), and an optimal cut-off value of OSI was 1.92, yielding a sensitivity of 0.70 and a specificity of 0.61.

## 4. Discussion

In this study, we evaluated the clinical utility of oxidative stress markers in patients with long COVID, particularly those infected during the Omicron phase. Serum levels of d-ROMs and OSI, but not BAP, were elevated in patients with long COVID compared with reference values derived from healthy individuals, with d-ROMs and OSI being notably higher in female patients with long COVID. Oxidative parameters were associated with age, BMI, inflammatory factors, and endocrine functions. Oxidative stress positively correlated with adrenocortical function and negatively correlated with thyroid function. Additionally, brain fog was significantly associated with elevated oxidative stress levels, especially in female patients with long COVID. Furthermore, ROC curve analysis demonstrated discriminative abilities for diagnosing long COVID and for identifying patients with brain fog among the patients with long COVID.

Fatigue, a major feature of long COVID, is a significant symptom with 46% of patients complaining of fatigue lasting for several months, and its pathophysiology has yet to be elucidated [22]. A more detailed characterization of post-COVID-19 fatigue requires multiple clinical and laboratory approaches to identify physical damage and mental health conditions. In addition to widely used screening tools for symptom differentiation [11,22], it has been revealed that oxidative stress markers might help distinguish acute, subacute, and resting fatigue in healthy individuals by comparing d-ROMs and BAP levels [30]. Furthermore, combining d-ROMs and BAP with OSI has proven useful in differentiating CFS from other fatigue-related conditions [30]. In that study, patients with CFS tended to exhibit higher d-ROM and OSI values and lower BAP values at rest than age- and sex-matched controls [30].

Although differentiating the causes of post-COVID-19 fatigue remains a clinical challenge, the measurement of d-ROMs and BAP may aid in distinguishing among various fatigue-related conditions in patients with long COVID. Our findings extend this application by demonstrating that these markers can also reflect brain fatigue, as evidenced by elevated d-ROMs and OSI in patients with long COVID experiencing brain fog. According to the Italian cohort study, the risk and prevalence of long COVID during the Omicron phase are highly affected by viral shedding from infections and vaccination status [11]. However, there were no significant differences in the levels of oxidative markers in our present long COVID patients with or without vaccinations.

In the present study, serum levels of d-ROMs and OSI were comparably higher than reference data from the healthy population, while the levels of BAP were moderately low compared to the healthy controls. In an earlier study of 312 healthy subjects, oxidative stress levels detected by OSI increased significantly with age [32], and the OSI levels were significantly higher in women compared to men [31]. The same trend was observed in the present study on patients with long COVID. Of note, serum BAP levels were found to be significantly decreased with age in patients with long COVID, suggesting the age-dependent impairment of antioxidative capacity in patients with long COVID.

One of the hallmarks of unexplained fatigue is ME/CFS, which includes symptoms such as pain, depression, and neurocognitive dysfunction. Although the etiology remains unclear, inflammation and oxidative/nitrosative stress are suspected contributors [39]. Nearly half of patients with long COVID meet the diagnostic criteria for ME/CFS [40,41], although the prevalence rates vary depending on the criteria applied. Our previous study showed that 8.4% of patients met the ME/CFS criteria [15], with a lower prevalence observed during the Omicron phase compared with earlier variants [42].

However, patients with long COVID during the Omicron phase reported brain fog more frequently [15], suggesting a variant-specific neurological effect [43]. This may involve direct viral entry into the central nervous system via angiotensin-converting enzyme 2 (ACE2) receptors in the alveolar epithelium, leading to disruption of the blood–brain barrier (BBB) and inflammation in the periventricular and choroid plexus regions [44,45,46]. These pathophysiological processes may explain the elevated oxidative stress and reduced antioxidant defenses observed in our study.

Recent investigations further provide indirect evidence that pathological fatigue is a neurological disorder, involving diminished muscular force and sensory attenuation [47]. Several studies support a relationship between brain fog and oxidative stress [48]. After SARS-CoV-2 infection, endothelial cells release pro-inflammatory cytokines and chemokines, which initiate cytokine storms and elevate reactive oxygen species (ROS) [49,50]. This leads to calcium imbalance and increased BBB permeability [51,52]. Viral binding to ACE2 receptors further disrupts renin-angiotensin homeostasis and enhances ROS production via NADPH oxidase [53]. Microglial activation and interleukin-1 up-regulation exacerbate oxidative damage, phagocytosis, and neural apoptosis [54]. These mechanisms may underlie vascular leakage and neuroinflammation [55], contributing to brain fog.

Endocrine functions may also affect the endogenous oxidative stress [56]. We previously reported that depression and fatigue conditions were correlated with adrenocortical and thyroid functions in patients with long COVID [27], in which the severity of infection and immunoreactions were associated with changes in HPA activity and thyroidal function [27]. These findings suggest that endocrine dysfunction is likely to be involved in the persistence of long COVID symptoms. Of interest, hypothyroidism has been linked to increased ROS, oxidative stress, and lipid peroxidation [57,58], while hyperthyroidism also shifts the pro-oxidant/antioxidant balance towards oxidative damage [56]. Our present results demonstrated that serum thyroxine levels inversely correlated with d-ROMs and OSI, suggesting a link between hypothyroidism and oxidative stress in long COVID. On the other hand, glucocorticoids (GCs) are known to promote neuronal oxidative stress by enhancing mitochondrial respiration and ROS generation [59]. Chronic GC excess is associated with various ROS-related pathologies, including myopathy, osteoporosis, diabetes, and hypertension [59]. The brain is particularly vulnerable to GC-induced oxidative damage owing to its high metabolic activity, low antioxidant capacity, and abundance of GC receptors [60], which may contribute to the linkage between oxidative stress and neurological symptoms in a state of hypercortisolemia. Although cortisol is known as a representative stress hormone, our study found that cortisol levels correlated with oxidative markers, indicating that hypercortisolemia may exacerbate endogenous oxidative stress in long COVID. Thus, our present results suggest that thyroid and adrenal dysfunctions exert opposing effects on redox balance in patients with long COVID.

This study has several limitations. First, it was a single-center, retrospective study conducted in Japan. As most participants were referred cases, patients with more severe long COVID symptoms may have been over-represented. Moreover, because this study was based in an outpatient clinic, individuals experiencing prolonged symptoms after severe acute COVID-19 may also have been over-represented. Information regarding the type and batch of mRNA vaccines administered was unavailable. Therefore, multicenter prospective studies are needed to validate these findings. Second, patient characteristics and comorbidities were not analyzed in detail. In addition, covariate data such as age, sex, and BMI were not available for the healthy control group, which made it impossible to perform fully adjusted multivariable analyses across patients and controls. We therefore conducted additional multivariable regression analyses within the patient group to account for patient background factors, but residual confounding cannot be fully excluded. Third, the timing and handling of blood samples varied and were not standardized across participants. Fourth, the cutoff values were determined in the laboratory based on previous reports by the co-authors, and no universally accepted standards have been established. Fifth, patients with non-specific symptoms showed OSI levels nearly overlapping with those without such symptoms, raising the possibility of false positives if OSI is used alone. These limitations should be addressed in future studies employing an appropriate study design.

## 5. Conclusions

In conclusion, oxidative stress markers, such as d-ROMs, BAP, and OSI, may serve as useful indicators for detecting or predicting neurologic symptoms of long COVID, particularly brain fog, in a gender-dependent manner. These markers may also be useful for assessing the pathophysiology and the severity of long COVID, especially when combined with inflammatory markers and endocrine data. Further studies are warranted to elucidate the oxidative stress–related pathophysiology underlying long COVID.

## Figures and Tables

**Table 1:** Clinical backgrounds of patients with long COVID enrolled in the present study.

Clinical Backgrounds of Long COVID (*n* = 77)	Patients’ Data
Median age (years) [IQR]	44 [32–58]
Sex, *n* (%)	
Male	36 (47)
Female	41 (53)
Median BMI [IQR]	23.3 [20.3–25.4]
Smoking habit, *n* (%)	
Yes	23 (30)
No	54 (70)
Severity of COVID-19 in acute phase, *n* (%)	
Mild	72 (94)
Moderate and Severe	5 (6)
Duration after the onset of COVID-19 to the first visit (%)	
≤90 days	28 (36)
>90 days	49 (64)
Vaccination, *n* (%)	
0 dose	12 (16)
≥2 doses	65 (84)

Medians [IQR] and percentages (%) are shown. BMI, body mass index; COVID-19, coronavirus disease 2019; IQR, interquartile range.

**Table 2:** Examined oxidative stress markers in patients with long COVID and healthy controls.

Oxidation Markers	Long COVID Patients	Control Group [31,32]	*p* Value
Case number: male/female Mean age (years)	77 cases: 36/53 45.4	312 cases: 148/164 36.7	
Median d-ROM [IQR] (CARR Unit)	533.8 [454.9–627.6]	287.4 [252.8–314.5]	<0.01
Median BAP [IQR] (μmol/L)	2385.8 [2169.2–2558.1]	2545.7 [2503.1–2583.5]	<0.01
Median OSI [IQR]	2.0 [1.7–2.5]	1.0 [0.9–1.1]	<0.01

BAP, biological antioxidant potential; d-ROMs, diacron-reactive oxygen metabolites; OSI, oxidative stress index; IQR, interquartile range. The Mann–Whitney U test was used for statistical comparisons.

**Table 3:** Oxidative stress markers and COVID-19 vaccination histories in patients with long COVID.

Oxidative stress Markers and Vaccination	0 Dose (*n* = 12)	≥2 Doses (*n* = 65)	*p* Value
Median d-ROM [IQR] (CARR Unit)	524.5 [461.4–623.8]	533.8 [435.5–627.6]	0.94
Median BAP [IQR] (μmol/L)	2361.9 [1971.6–2607.7]	2385.8 [2214.9–2556.5]	0.68
Median OSI [IQR]	2.1 [1.7–2.4]	2.0 [1.7–2.5]	0.88

BAP, biological antioxidant potential; d-ROMs, diacron-reactive oxygen metabolites; OSI, oxidative stress index; IQR, interquartile range. The Mann–Whitney U test was used for statistical comparisons.

**Table 4:** Multivariable linear regression analysis of factors associated with OSI in patients with long COVID.

Variables	β-Coefficient	95% CI	*p*-Value
Age (per year increase)	0.007	−0.0004–0.014	0.063
Male sex (vs. Female)	−0.322	−0.594–0.050	0.021
BMI (per 1 kg/m^2^ increase)	0.032	0.002–0.063	0.039

CI: confidence interval. Multivariable linear regression analyses were conducted within the patient group.

## Data Availability

Detailed data are available upon request from the corresponding author. The data are not publicly available due to the ethical reason.

## Abbreviations

angiotensin-converting enzyme 2 (ACE2), biological antioxidant potential (BAP), blood-brain barrier (BBB), body mass index (BMI); coronavirus disease 2019 (COVID-19); COVID-19 aftercare clinic (CAC); diacron-reactive oxygen metabolites (d-ROMs), glucocorticoids (GCs), hypothalamo-pituitary-adrenal (HPA), myalgic encephalomyelitis/chronic fatigue syndrome (ME/CFS); oxidative stress index (OSI); post-COVID-19 condition (PCC), reactive oxygen species (ROS).
